# Acetaldehyde inhibits retinoic acid biosynthesis to mediate alcohol teratogenicity

**DOI:** 10.1038/s41598-017-18719-7

**Published:** 2018-01-10

**Authors:** Yehuda Shabtai, Liat Bendelac, Halim Jubran, Joseph Hirschberg, Abraham Fainsod

**Affiliations:** 1 Department of Developmental Biology and Cancer Research, Institute for Medical Research Israel-Canada, The Hebrew University of Jerusalem, Jerusalem, Israel; 2 Department of Genetics, The Alexander Silberman Institute of Life Sciences, Faculty of Science, The Hebrew University of Jerusalem, Jerusalem, Israel

## Abstract

Alcohol consumption during pregnancy induces Fetal Alcohol Spectrum Disorder (FASD), which has been proposed to arise from competitive inhibition of retinoic acid (RA) biosynthesis. We provide biochemical and developmental evidence identifying acetaldehyde as responsible for this inhibition. In the embryo, RA production by RALDH2 (ALDH1A2), the main retinaldehyde dehydrogenase expressed at that stage, is inhibited by ethanol exposure. Pharmacological inhibition of the embryonic alcohol dehydrogenase activity, prevents the oxidation of ethanol to acetaldehyde that in turn functions as a RALDH2 inhibitor. Acetaldehyde-mediated reduction of RA can be rescued by RALDH2 or retinaldehyde supplementation. Enzymatic kinetic analysis of human RALDH2 shows a preference for acetaldehyde as a substrate over retinaldehyde. RA production by hRALDH2 is efficiently inhibited by acetaldehyde but not by ethanol itself. We conclude that acetaldehyde is the teratogenic derivative of ethanol responsible for the reduction in RA signaling and induction of the developmental malformations characteristic of FASD. This competitive mechanism will affect tissues requiring RA signaling when exposed to ethanol throughout life.

## Introduction

Alcohol (ethanol; EtOH) exposure of human embryos results in a complex set of anatomical, mental and behavioral abnormalities collectively known as Fetal Alcohol Spectrum Disorder (FASD)^1,2^. Children with FASD can exhibit facial dysmorphology, microcephaly, short stature, central nervous system and neurodevelopmental abnormalities including intellectual disabilities and behavioral and psychological problems. The incidence of FASD can reach 2–5% of school children^3–6^. Oxidation of EtOH to acetaldehyde (AcAL) is the first step in the alcohol detoxification process^7^. This reaction is performed mainly by members of the alcohol dehydrogenase (ADH) enzyme family, peroxisomal catalase and the cytochrome P450 CYP2E1^8^. Efficient oxidation of AcAL to acetate is of great importance for its elimination due to its high toxicity. In adults, this oxidation reaction is mainly performed by members of the aldehyde dehydrogenase family, like; ALDH2, ALDH1B1, and ALDH1A1^8^.

One of the models to explain the etiology of FASD, proposed an inhibitory effect on retinoic acid (RA) biosynthesis^9–11^. RA is produced from vitamin A (retinol, ROL) first by alcohol dehydrogenase (ADH), or short-chain dehydrogenase/reductase (SDR)-mediated oxidation to produce retinaldehyde (RAL). A subsequent oxidation step from RAL to RA is performed by aldehyde dehydrogenases^12,13^. The EtOH/RA competition model suggested that the enzymatic overlap between EtOH detoxification and RA biosynthesis results in competitive inhibition. RA performs numerous regulatory functions during embryogenesis and adult tissue homeostasis, including tumor suppression. Therefore, its levels and localization are continuously regulated, and deviation from normal physiological levels results in multiple, and sometimes severe developmental malformations^14–16^.

Developmental biochemical characterization of the competition model during early embryogenesis, showed that EtOH exposure reduced RA signaling, affected known RA-regulated genes, and induced phenotypes recapitulating the malformations characteristic of FASD^17^. Of particular importance was the demonstration that ROL or RAL supplementation of EtOH-treated embryos^18^ or increasing the retinaldehyde dehydrogenase activity^19^ can rescue the abnormally low RA signaling levels, restore normal gene expression and prevent the characteristic developmental malformations. In agreement, retinaldehyde dehydrogenase 2 (RALDH2; ALDH1A2) knockdown resulted in enhanced sensitivity to alcohol^19^. As AcAL is oxidized to acetic acid by an ALDH enzyme, and RALDH2 is the earliest zygotic RALDH (ALDH) expressed in the embryo^20^, these results suggested that RALDH2 is probably the earliest enzymatic activity competed by EtOH, resulting in the inhibition of RA biosynthesis.

In this study, we elucidated the etiology of ethanol in FASD during early embryogenesis, as an inhibitor of RA biosynthesis. We demonstrate that in early embryogenesis, both EtOH and its oxidation product, AcAL, similarly repress RA signaling. Importantly, during early development, the RA-inhibitory effect of EtOH is dependent on its oxidation to AcAL by ADH. Furthermore, we show that EtOH and AcAL inhibit the activity of human RALDH2 (hRALDH2) *in vivo*. Surprisingly, the kinetic analysis revealed AcAL to be a preferred substrate of hRALDH2 over RAL, while EtOH is not an inhibitor of this enzyme. Our study elucidates the mechanism of RA inhibition during early embryogenesis and suggests possible roles for EtOH in some types of tumors.

## Results

### ADH activity is required to convert EtOH to its teratogenic derivative

**Figure 1:**
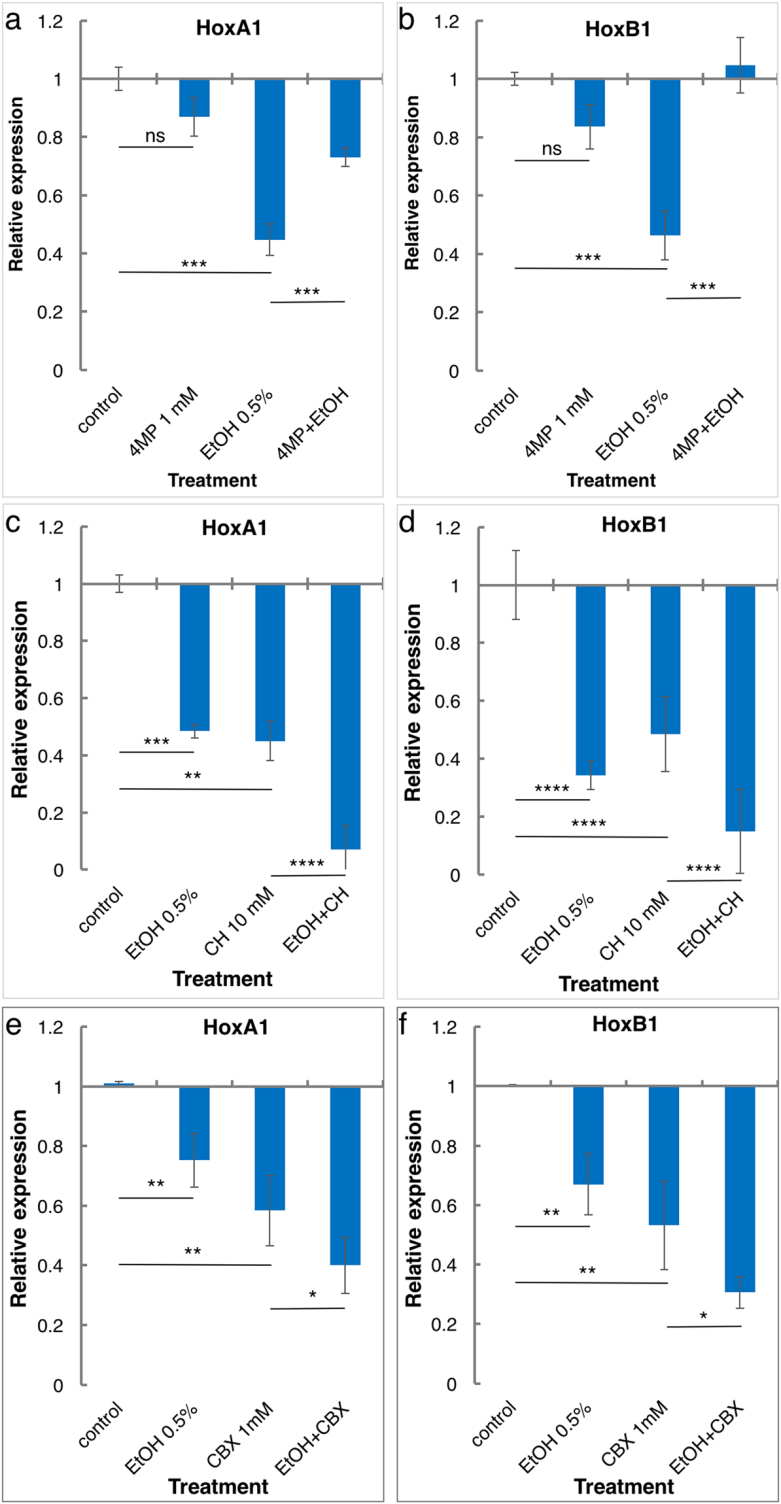
Ethanol-dependent retinoic acid inhibition requires middle-chain alcohol dehydrogenases. The enzymatic requirements for EtOH to inhibit RA biosynthesis were studied using inhibitors of the middle-chain alcohol dehydrogenases (ADH), 4-methylpyrazole (4MP), or the short-chain dehydrogenase/reductases, carbenoxolone (CBX) and chloral hydrate (CH). Late blastula stage embryos were treated with EtOH alone or in combination with 4MP (**a**,**b**), CH (**c**,**d**) or CBX (**e**,**f**). The effect on RA signaling was determined by monitoring the expression level of the known RA-regulated genes, *HoxA1* and *HoxB1* during early gastrula stages. n = 3, The values denote mean ± SEM. P values - *p < 0.05; **p < 0.01; ***p < 0.001; ****p < 0.0001; ns, not significant.

To analyze the competition of EtOH for the ADH enzymes available as proposed by the RA biosynthesis inhibition model, we established the conditions for their inhibition using 4-methylpyrazole (4MP)^21^. Late blastula (st. 8.5) *Xenopus* embryos were treated with increasing concentrations of 4MP (1 µM–1 mM) and the effect on RA levels was studied during early/mid-gastrula (st. 10.5). We monitored the expression of the RA target genes, *HoxA1* and *HoxB1* ^22^ by quantitative real-time PCR (qPCR). Inhibition of the ADH activity only had a very mild repressive effect on *Hox* expression (Supplementary Fig. S1a,b) suggesting that 4MP at these concentrations does not affect the production of RA. Taking advantage of 4MP, we investigated whether EtOH hampers RA biosynthesis, or it has to be oxidized to AcAL to achieve its teratogenic effect (Fig. 1a,b and Supplementary Fig. S2a,b). EtOH alone (0.5% v/v; 86 mM) induced a strong reduction (about 50% inhibition) in *HoxA1, HoxB1*, *Dhrs3* and *Cyp26A1* expression. This expression repressive effect of EtOH was blocked by 4MP (1 mM) supporting the requirement to oxidize EtOH to AcAL by members of the ADH family.

Oxidation of ROL to RAL during gastrula stages has been attributed to retinol dehydrogenases (RDH) of the SDR family^23,24^. To study the role of SDRs in EtOH teratogenesis, we employed chloral hydrate (CH)^25^ and carbenoxolone (CBX)^26^ as RDH inhibitors. Late blastula embryos were treated with 5–30 mM CH or 10 µM to 1 mM CBX exhibited a reduction in *Hox* expression (Supplementary Fig. S1c–f). Inhibition of the RDH activity with 10 mM CH or 1 mM CBX repressed *HoxA1, HoxB1*, *Dhrs3* and *Cyp26A1* expression by over 50% (Fig. 1c,d and Supplementary Fig. S2c,d). EtOH treatment (86 mM), had a similar repressive effect which was not affected by RDH inhibition and they behaved additively reaching about 90% inhibition of gene expression (Fig. 1c,d and Supplementary Fig. S2c,d). Also, 1 mM CBX did not inhibit the *Hox* repressive effect of EtOH (Fig. 1e,f). We conclude that, while the RDH is central for the oxidation of ROL to RAL in early embryos, enzymes of the ADH family are required for the oxidation of EtOH to its RA-repressive metabolite, acetaldehyde.

### Acetaldehyde recapitulates the teratogenic effects of ethanol

To determine the effect of AcAL exposure on RA-regulated genes, embryos were treated with various acetaldehyde concentrations in the physiological range^27–31^. Embryos were exposed to AcAL (1–10 µM) from late blastula (st. 8.5), and the expression levels of several RA-regulated genes was analyzed by qPCR during early/mid-gastrula (st. 10.5). The expression of *HoxA1*, *HoxB1*, *HoxB4*, *Dhrs3, Cyp26A1*, and *Rdh10* was affected by the AcAL treatment such that *Hox* gene expression was reduced in a concentration-dependent fashion, while *Cyp26A1* and *Dhrs3* showed repression and *Rdh10* was up-regulated (Supplementary Fig. S3).

**Figure 2:**
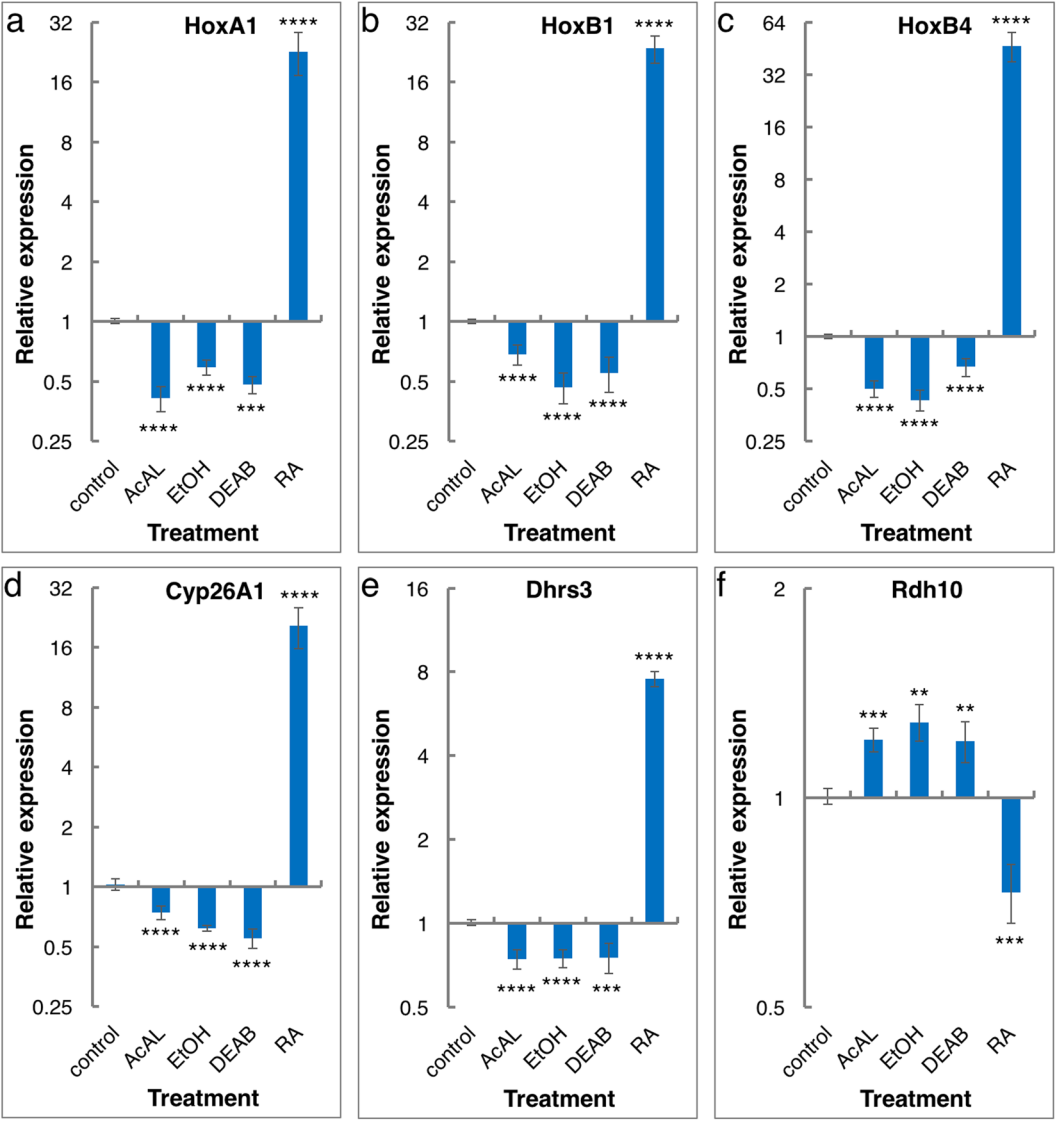
Acetaldehyde induces a reduction in retinoic acid signaling. The changes in the expression of several RA-regulated genes was determined after AcAL (5 µM), EtOH (0.5%), DEAB (100 µM), or RA (1 µM). Embryos were treated during late blastula (8.5), and RNA was extracted for analysis during early/mid-gastrula (st. 10.5). The expression level of the RA-regulated genes *HoxA1* (**a**), *HoxB1* (**b**), *HoxB4* (**c**), *Cyp26A1* (**d**), *Dhrs3* (**e**), and *Rdh10* (**f**) by qPCR. n = 4, The values denote mean ± SEM. P values - **p < 0.01; ***p < 0.001; ****p < 0.0001.

To further link acetaldehyde to the EtOH-induced reduction of RA levels, the expression of the RA-regulated genes was studied in embryos treated in parallel with AcAL (5 µM), EtOH (0.5%), the RALDH inhibitor, 4-diethylaminobenzaldehyde (DEAB, 100 µM)^32^ and RA (1 µM). Acetaldehyde treatment induced a 30% to 60% reduction or increase in expression depending on the gene in question and in accordance with its normal response to RA signaling (Fig. 2). The EtOH and DEAB treatments resulted in very similar effects on gene expression, consistent with AcAL inducing a reduction in RA signaling. In contrast, RA exposure induced the expected opposite effect (Fig. 2).

**Figure 3:**
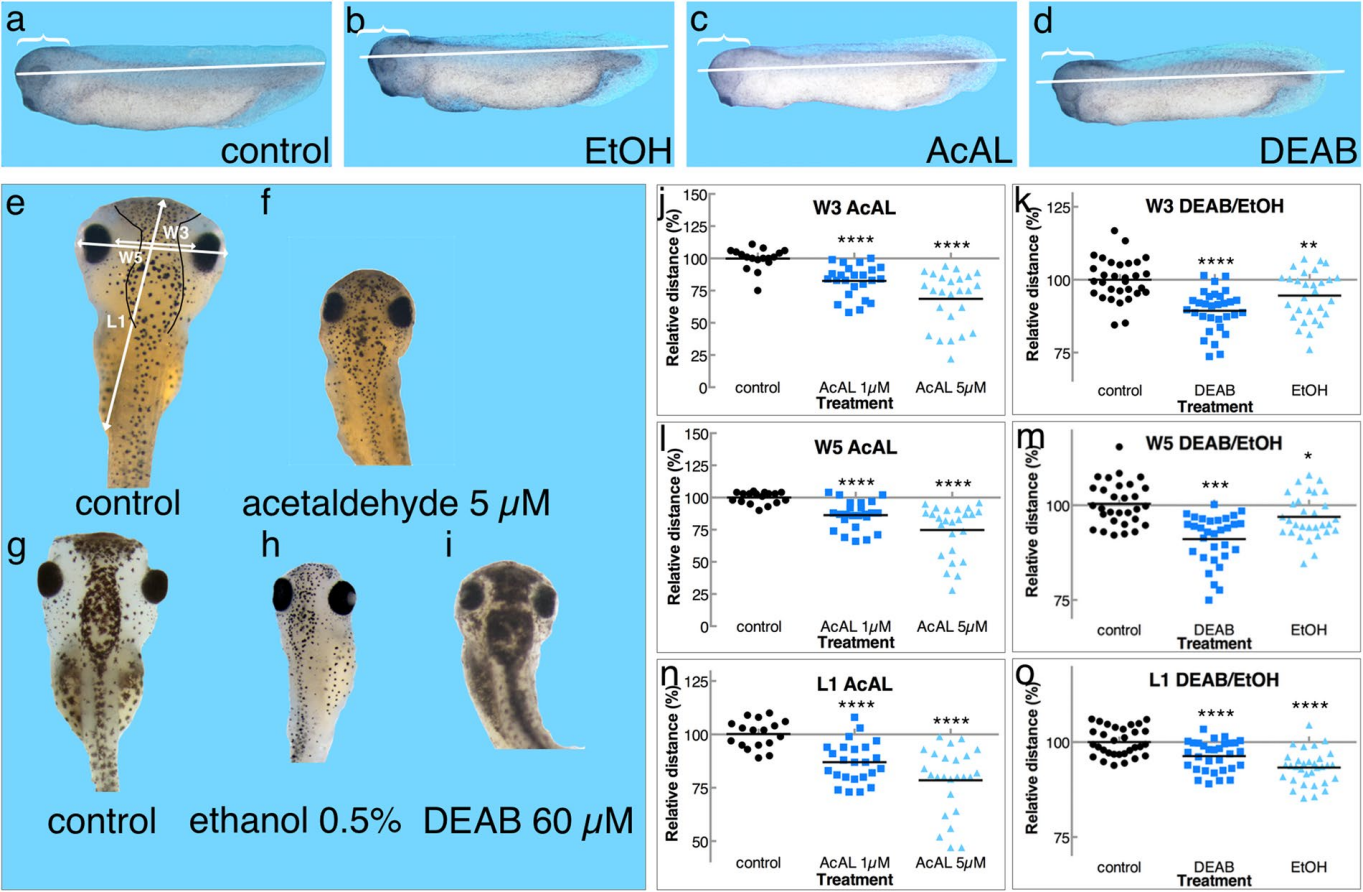
Acetaldehyde phenocopies the malformations induced by ethanol. Embryos were treated with AcAL (5 µM), EtOH (0.5%) or DEAB (60 µM) and allowed to develop. At stage 34, embryos were assessed for general developmental malformations comparing controls (**a**) to EtOH (**b**), AcAL (**c**) and DEAB (**d**) treated embryos. For better qualitative length comparison, a line was drawn from the forehead to the tail-tip of the control embryo (**a**). This line was copied unto the treated embryos (**b**,**c**,**d**). For head size comparison, a bracket was drawn from the forehead to the beginning of the dorsal fin (**a**). A copy of the same bracket was placed at the onset of the dorsal fin in the treated embryos (**b**,**c**,**d**). For a more quantitative comparison of the malformations, at stage 45 the malformations in head formation were characterized. (**e**–**i**) The head region of control (**e** and **g**), AcAL (**f**), EtOH (**h**) and DEAB (**i**) treated embryos. The head anatomical distances measured according to Nakatsuji^33^ are shown. W3, inner distance between eyes; W5, head width; L1, length of head. Head malformations are shown as changes in size from control values for all the parameters, n = 70 (**j**–**o**). Two AcAL concentrations (1 µM and 5 µM) are shown (**j**, **l** and **n**). The size changes for EtOH and DEAB treated embryos are shown, n = 94 (**k**, **m** and **o**). P values - *p < 0.05; **p < 0.01; ***p < 0.001; ****p < 0.0001.

To show that acetaldehyde recapitulates the developmental malformations induced by ethanol^33^, we analyzed the developmental defects resulting from these treatments (Fig. 3). Embryos were treated with AcAL (5 µM), EtOH (0.5%), or DEAB (60 µM) and allowed to develop to st. 34 (Fig. 3a–d). All three treatments affected the formation of the head, the eye, the trunk, and the tailbud (Fig. 3a–d). The length of the embryos was determined for all samples and it was found that there is a significant shortening in the treated embryos compared to controls (Fig. 3a–d). A more detailed and quantitative comparison between these treatments, was performed on a second set of treated embryos allowed to develop to stage 45. The distance between eyes (W3,) head width (W5), and head length (L1) were measured according to Nakatsuji^32^. All head parameters were significantly reduced in AcAL-treated embryos (Fig. 3f,j,l,n) and the effects were similar to those induced by EtOH treatment or down-regulation of RA biosynthesis (DEAB; Fig. 3h,i,k,m,o). These results show that AcAL, as the intermediate in EtOH detoxification, induces the same set of molecular and developmental defects as the actual alcohol exposure.

One striking effect of EtOH exposure is a reduction in brain size^34^. To characterize the brain malformations resulting from AcAL-exposure, embryos were treated with AcAL (5 µM) or EtOH (0.5%), and the relative position of rhombomere 5 (R5) and the midbrain-hindbrain boundary (MHB) were determined using *HoxB3* and *en2* as markers, respectively (Supplementary Fig. S4). Measurement of distance from the rostral end of the neural plate (RNP) to the MHB or from the MHB to R5 revealed a statistically significant reduction of about 15% in brain size (Supplementary Fig. S4).

### Acetaldehyde competes with retinaldehyde for the available RALDH2 activity

**Figure 4:**
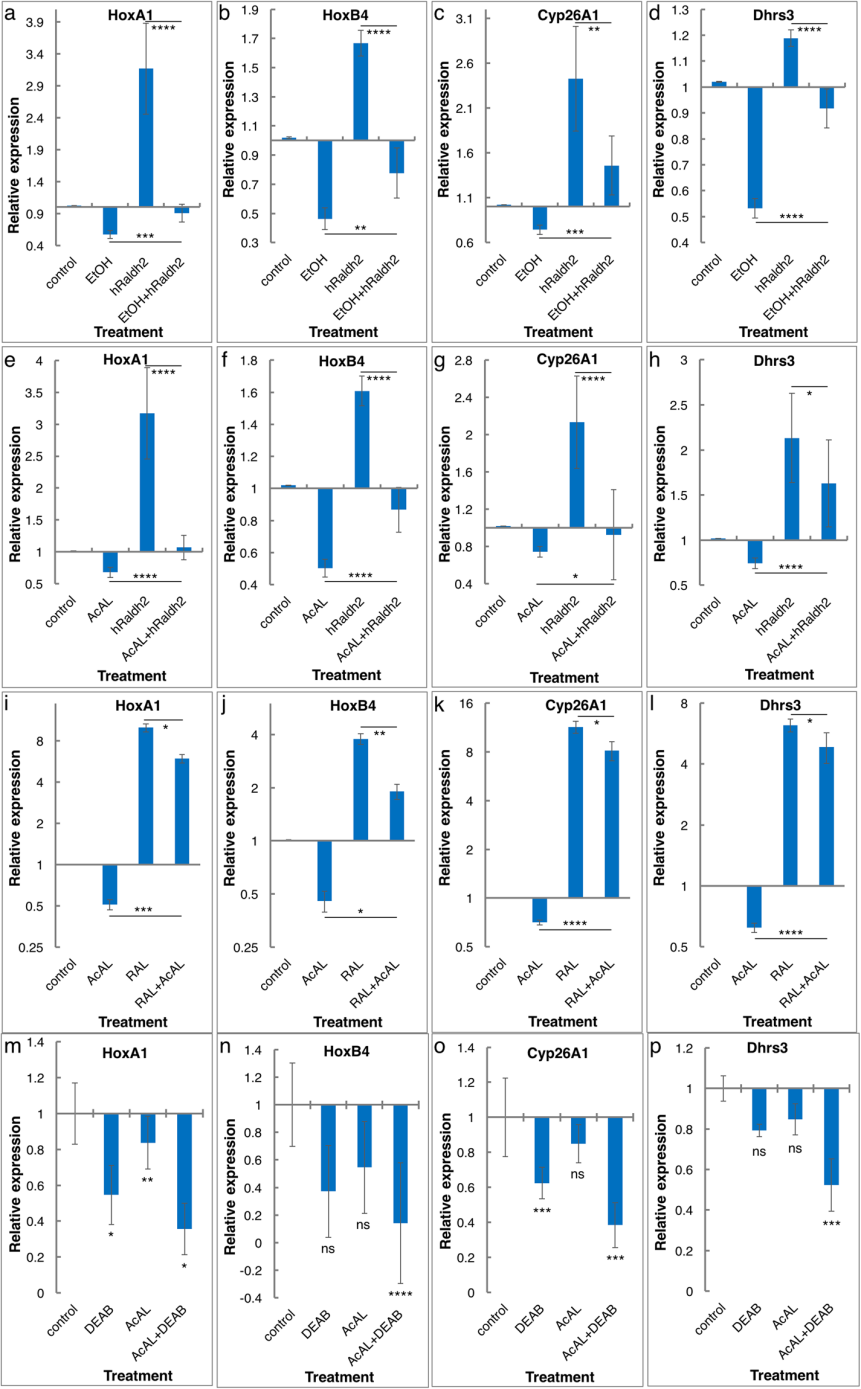
Acetaldehyde competes for the human RALDH2. The effect of ethanol and its oxidation product, acetaldehyde, on hRALDH2 activity was studied *in vivo*. Embryos injected with plasmid encoding hRALDH2 or control embryos were treated with AcAL (5 µM), EtOH (0.5%), RAL (1 µM) or DEAB (20 µM), individually or in different combinations. Treatments were initiated during late blastula, and RNA samples were prepared during early/mid-gastrula. The effect of the combined treatments was determined by analyzing the response of the RA-regulated genes, *HoxA1* (**a**,**e**,**i**, and **m**), *HoxB4* (**b**,**f**,**j** and **n**), *Cyp26A1* (**c**,**g**,**k** and **o**) and *Dhrs3* (**d**,**h**,**l** and **p**) by qPCR. (**a**–**d**) Overexpression of hRALDH2 together with EtOH treatment. (**e**–**h**) AcAL treatment together with hRALDH2 overexpression. (**i**–**l**) Combined treatment with AcAL and RAL. (**m**–**o**) Combined treatment with DEAB and AcAL. n = 3, The values denote mean ± SEM. P values - *p < 0.05; **p < 0.01; ***p < 0.001; ****p < 0.0001; ns, not significant.

We previously showed that the embryonic, RALDH2-dependent RA production is sensitive to EtOH^19^. To determine whether EtOH also inhibits the human RALDH2 enzyme, embryos injected with a *hRaldh2* expression plasmid were exposed to EtOH from the midblastula transition and analyzed during early/mid-gastrula (st. 10.5). *hRaldh2* overexpression resulted in the expected gene-dependent up-regulation (*HoxB1*, *HoxA1*, *HoxB4*, *Dhrs3*, and *Cyp26A1*) and down-regulation (*Rdh10*) in expression supporting the conclusion that this enzyme increases the endogenous RA (Fig. 4a–d and Supplementary Fig. S5a,b). EtOH treatment induced the opposite effect on gene expression consistent with a reduction in RA levels, while a combination of both treatments had a rescuing effect (Fig. 4a–d and Supplementary Fig. S5a,b). We conclude that hRALDH2 can produce RA and this activity is susceptible to EtOH-promoted inhibition.

To show that this conclusion also holds true for acetaldehyde, embryos were injected with the *hRaldh2* expression plasmid and treated with AcAL. Acetaldehyde treatment alone induced the expected abnormal expression of RA-regulated genes (*HoxB1*, *HoxA1*, *HoxB4*, *Dhrs3*, *Cyp26A1*, and *Rdh10*) like EtOH exposure consistent with a reduction in RA signaling (Fig. 4e–h and Supplementary Fig. S5c,d). Increasing the amount of RALDH2 activity had a rescuing effect and partially restored normal expression levels. These results further support the effect of AcAL on RA signaling through the competition for the available RALDH activity *in vivo*.

**Figure 5:**
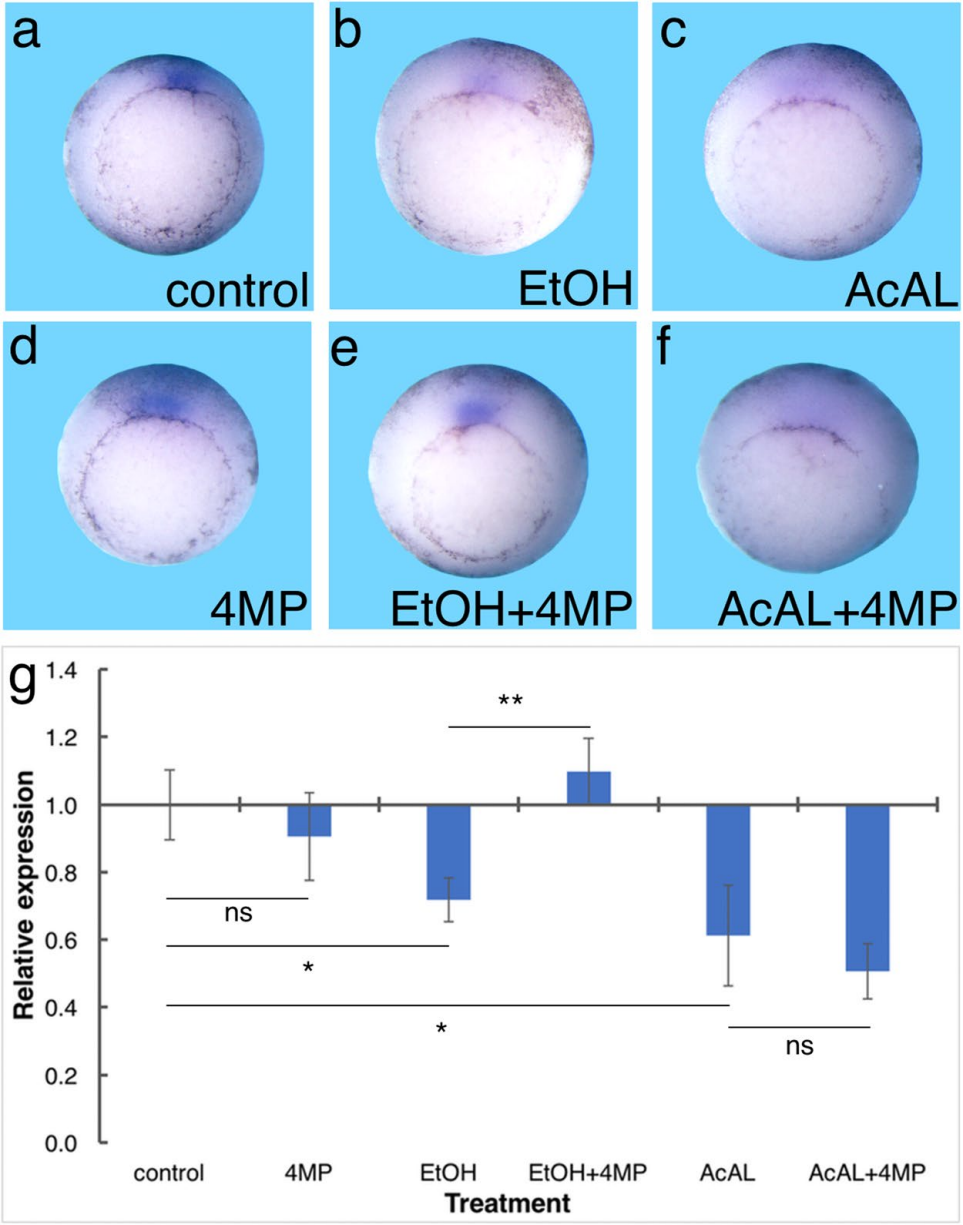
Acetaldehyde inhibits the expression of organizer-specific genes. Embryos were treated with EtOH (0.5%) or AcAL (5 µM) alone or together with 4MP (1 mM) to inhibit the ADH activity. (**a**–**f**) *In situ* hybridization analysis of the effects of EtOH and AcAL treatments on *gsc* expression. (**a**) control, (**b**) EtOH, (**c**) AcAL, (**d**) 4MP, (**e**) EtOH + 4MP and (**f**) AcAL + 4MP treated embryos. (**g**) qPCR analysis of the effect of the same treatments on *chordin* expression. n = 3, The values denote mean ± SEM. P values - *p < 0.05; **p < 0.01; ns, not significant.

These results and the use of 4MP inhibition support the conclusion that EtOH is converted to AcAL by enzymes of the ADH family and AcAL in turn competes for the RALDH activity. Previously we have shown that during early gastrula, organizer-specific genes like *gsc* and *chordin* are affected by EtOH and RA^18^. To further support the necessity to oxidize EtOH to AcAL to induce the detrimental effects on gene expression, embryos were treated with the ADH inhibitor 4MP. Both, EtOH and AcAL at physiological concentrations reduced the expression of *gsc* and *chordin* (Fig. 5b,c,g). The inclusion of 4MP in these treatments rescued the detrimental effects of EtOH (Fig. 5e,g) but had no influence on the gene expression effect of the AcAL treatment (Fig. 5f,g) further supporting the teratogenic role of AcAL.

To provide *in vivo* evidence of the acetaldehyde/retinaldehyde competition, embryos were treated with AcAL (5 µM), RAL (1 µM) or a combination of both, and the expression of known RA-regulated genes was determined. This type of analysis shows that RAL supplementation results in effects consistent with increased RA, while AcAL treatment induces the opposite response in agreement with a reduction in RA levels (Fig. 4i–l and Supplementary Fig. S5e,f). Moreover, combined treatment has a rescuing effect on RA target gene expression. These results suggest that increased RAL levels can rescue the teratogenic effects of acetaldehyde.

Further support for the involvement of a RALDH enzyme, probably RALDH2 at these stages, in the AcAL-induced reduction in RA levels was obtained using DEAB. According to our previous observations^18^, embryos simultaneously treated with DEAB and AcAL should exhibit a stronger reduction in RA signaling levels due to a double inhibition of the RALDH enzymatic activity Analysis of the expression of RA-regulated genes (*HoxB1*, *HoxB4*, *Cyp26A1* and *Dhrs3*) revealed lower expression levels in the combined treatment than in each of the individual treatments (Fig. 4m–p).

### Acetaldehyde is an efficient substrate of RALDH2

**Figure 6:**
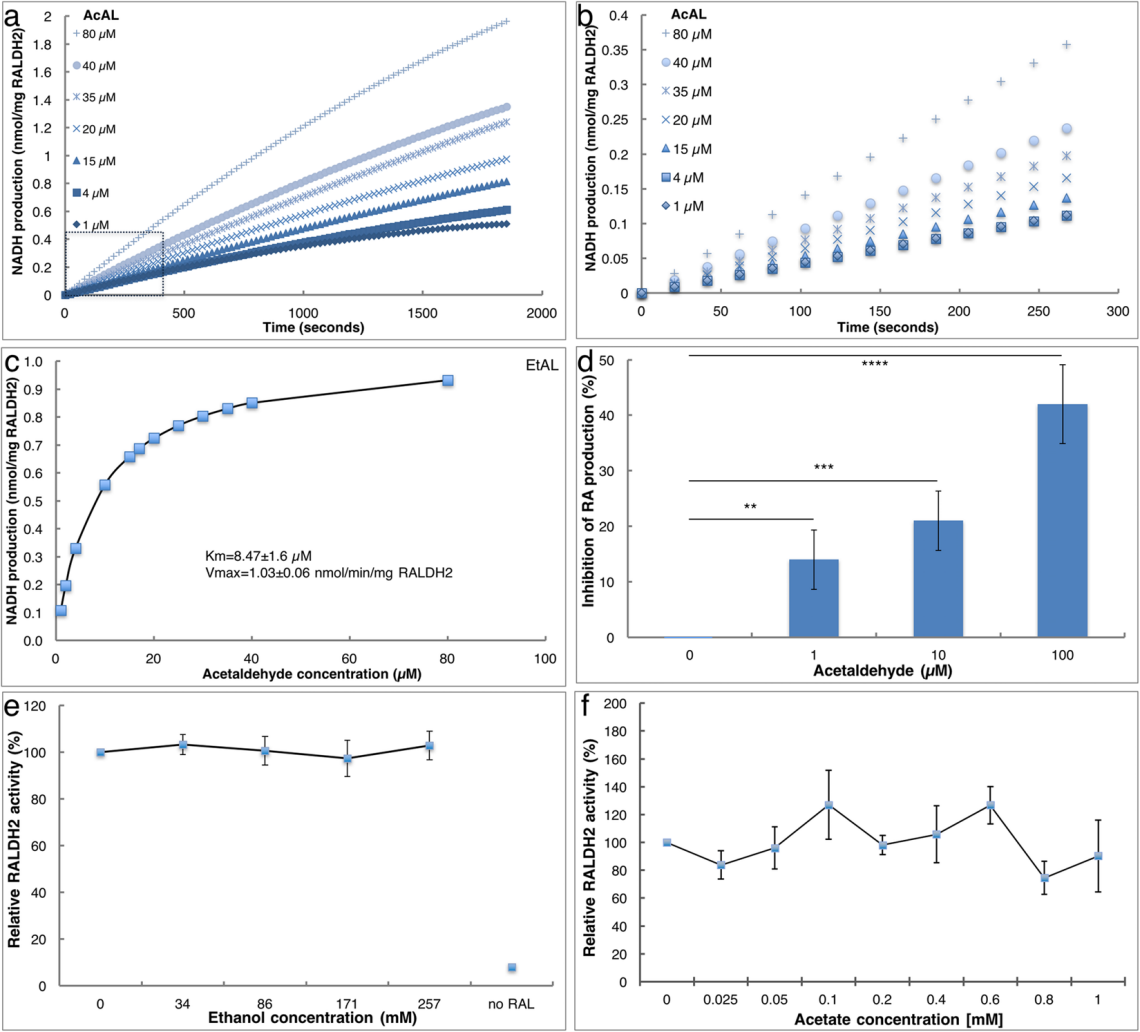
Acetaldehyde inhibits RA production by human RALDH2. The effect of ethanol and its oxidation product, acetaldehyde, was studied *in vitro* using a recombinant hRALDH2. (**a**,**b**) Increasing concentrations of EtOH covering the physiological range and above were added to RAL oxidation reactions *in vitro*. The efficiency of RAL oxidation was compared to the control sample without EtOH. (**a**) Kinetic analysis of the AcAL titration to determine the linear range of the oxidation reaction. (**b**) The initial 270 seconds of the reaction with different AcAL concentrations are shown. (**c**) Michaelis-Menten plot of the AcAL oxidation reaction by hRALDH2. (**d**) Inhibition of RAL oxidation to RA by increasing of acetaldehyde concentrations. The production of RA was determined by HPLC. (**e**) hRALDH2 activity in the presence of increasing EtOH concentrations. (**f**) The effect of increasing acetate concentrations on the activity of hRALDH2. n = 3, P values - **p < 0.01; ***p < 0.001; ****p < 0.0001.

The results obtained by analyzing RA-dependent gene expression *in vivo* supported the conclusions: i) AcAL recapitulates all the molecular and developmental effects originally attributed to EtOH, ii) the effect of AcAL is mainly mediated through the inhibition of RA biosynthesis, and finally, iii) AcAL is produced through ADH-mediated oxidation of EtOH. To biochemically demonstrate the competition between RAL and AcAL for the RALDH activity, we established *in vitro* enzyme assays with a recombinant hRALDH2 enzyme^35^. To test whether acetaldehyde might function as a competing substrate of hRALDH2, we studied the oxidation of AcAL. Using the previously described reaction conditions^35^ we tested increasing concentrations of AcAL (Fig. 6a,b). At low concentrations (1–80 µM), the reactions reached substrate depletion after 30 min (Fig. 6a) but, during the first 5 minutes, all reactions behaved linearly and showed a clear concentration dependence (Fig. 6b). Michaelis-Menten analysis of the reaction kinetics gave a K_m_ of 8.47 ± 1.6 µM and a V_max_ of 1.03 ± 0.06 nmol/min/mg hRALDH2 for acetaldehyde (Fig. 6c). The K_m_ for AcAL is about half of that for RAL, with very similar V_max_ for both substrates^35^, suggesting a hRALDH2 preference for AcAL.

To clearly demonstrate the competition between RAL and AcAL for hRALDH2, we analyzed the reaction progression by HPLC to directly determine the oxidation of RAL to RA in the presence of AcAL. Oxidation reactions of RAL by hRALDH2 were performed with increasing concentrations of AcAL (1–100 µM). At different times points, samples were collected, and the production of RA was quantitated. The addition of AcAL to the reaction mixture resulted in a concentration-dependent inhibition of the oxidation of RAL to RA (Fig. 6d) demonstrating the competition between RAL and AcAL for the available hRALDH2.

To rule out an inhibitory effect of EtOH on the activity of hRALDH2, the oxidation of *all-*trans retinaldehyde (RAL) was performed in the presence of increasing concentrations of EtOH. EtOH was increased for up to three times the maximal concentration in human blood. At all EtOH concentrations, the oxidation of RAL was not affected by the presence of EtOH (up to 257 mM) showing that it has no effect on the activity of hRALDH2 (Fig. 5e). Similarly, acetic acid (25 µM-1 mM), the oxidation product of AcAL did not affect the hRALDH2 activity (Fig. 6f).

### RALDH2 is the most abundant ALDH expressed during early gastrula

**Figure 7:**
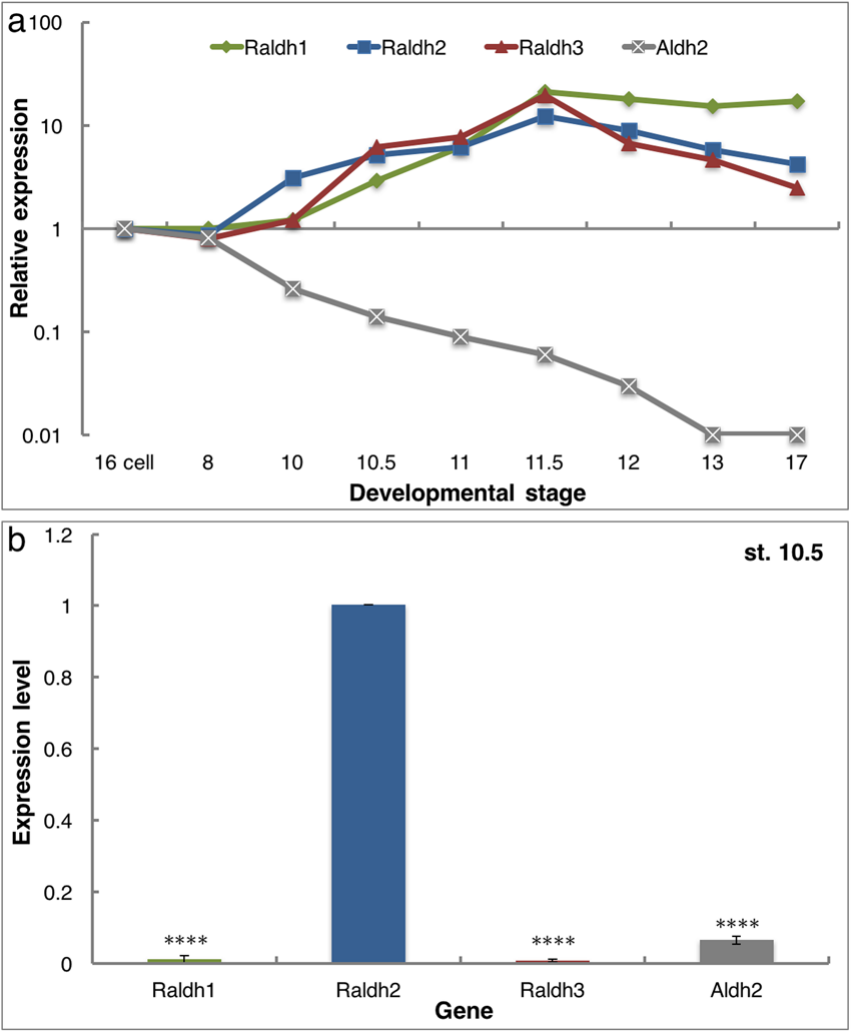
RALDH2 is the main RA-producing enzyme in the gastrula embryo. (**a**) Temporal pattern of expression of *Raldh1* (*Aldh1A1*), *Raldh2* (*Aldh1A2*), *Raldh3* (*Aldh1A3*) and *Aldh2*. RNA samples were collect from embryos from the 16-cell stage to neurula stages (st. 17). Expression levels were normalized to the 16-cell RNA amounts. (**b**) Relative transcript abundance during early/mid gastrula (st. 10.5). The relative expression of the different aldehyde dehydrogenases was calculated relative to the level of *Raldh2*. n = 3, The values denote mean ± SEM. P values - ****p < 0.0001.

The identification of acetaldehyde as the teratogenic metabolite of ethanol exposure prompted us to understand the ability of the early embryo to oxidize AcAL, a reaction efficiently performed by mitochondrial ALDH2 in the adult liver^36,37^. To determine whether ALDH2 is available in the embryo, the temporal pattern of *Aldh2* expression was studied by qPCR. The results show that *Aldh2* transcripts are maternally deposited in the oocyte, but their level decreases steeply, over 90% reduction, towards the onset of gastrulation becoming almost absent (Fig. 7a). These results show that during the high EtOH sensitivity window, from MBT until mid-gastrulation^18^, *Aldh2* expression is very low. As our results support a competition between AcAL and RAL, we also determined the temporal expression pattern of the three known *Raldh* genes: *Raldh1* (*Aldh1A1*), *Raldh2* (*Aldh1A2*) and *Raldh3* (*Aldh1A3*). Although all three genes are expressed zygotically, *Raldh2* is the earliest transcribed from midblastula (st. 8.5), while *Raldh1* and *Raldh3* expression only starts with the onset of gastrulation (Fig. 7a).

These expression patterns raise the possibility that any RALDH could be a target for AcAL competition. For this reason, we determined the RALDH relative abundance during early gastrula (st. 10.5). We found that the transcript levels of *Aldh2, Raldh1*, and *Raldh3* are 0.06, 0.01, and 0.01-fold lower respectively relative to the expression of *Raldh2* (Fig. 7b). This result places *Raldh2* as the major RA-producing enzyme at this developmental stage, but also as the primary target for acetaldehyde due to low quantities of ALDH2 in the early embryo.

## Discussion

**Figure 8:**
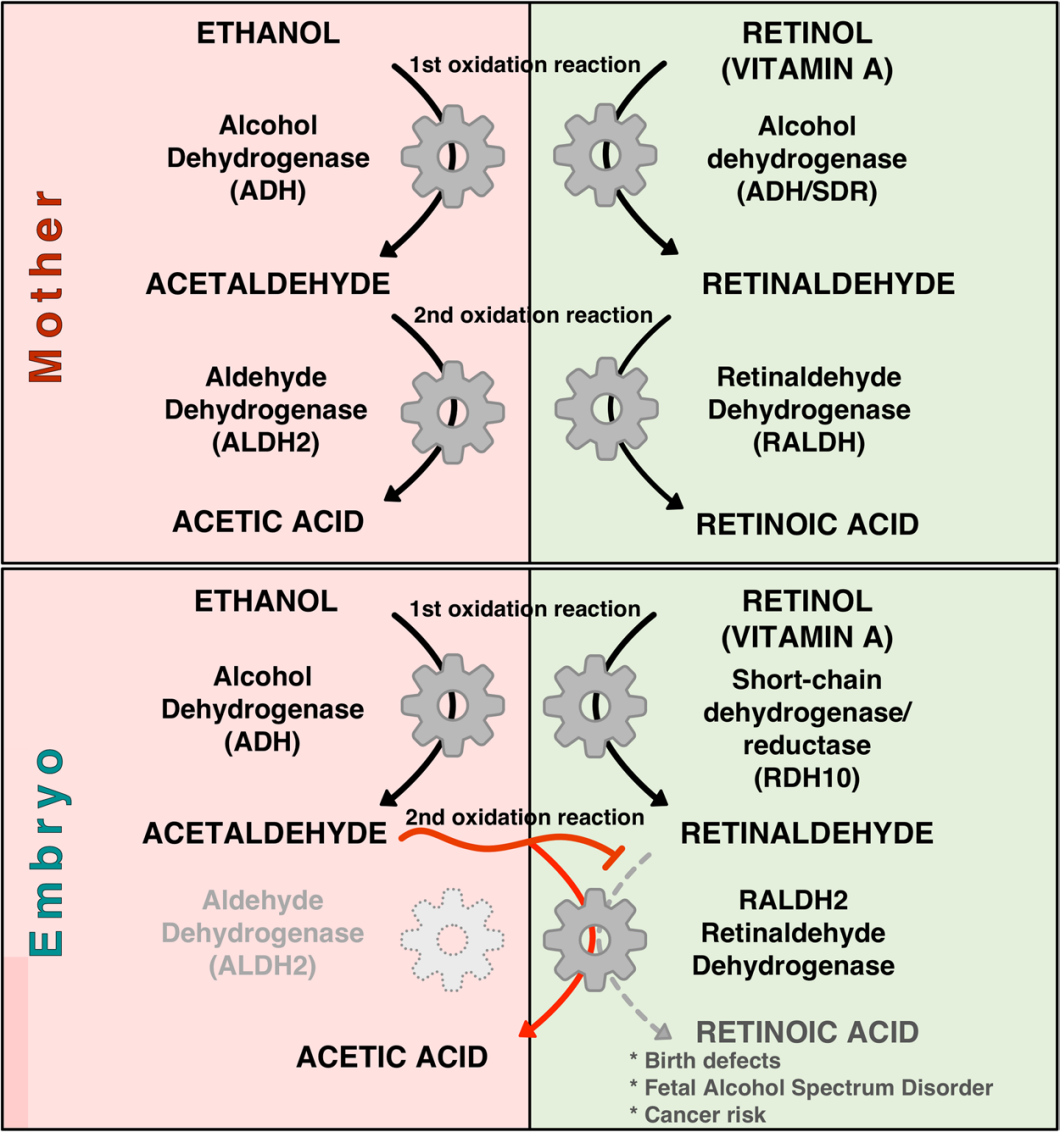
Biochemical competition between ethanol detoxification and retinoic acid biosynthesis. Schematic model of the enzymatic activities in humans involved in ethanol detoxification (left panels) and retinoic acid biosynthesis (right panels). The enzymes active in the mother are shown in the upper panel and the lower panel shows the enzymatic activities in the developing fetus.

One of the models proposed to explain the teratogenic effects of EtOH was based on biochemical knowledge, and it suggested that the ethanol detoxification competes with ROL for the available alcohol-oxidizing enzymatic activities also necessary for RA biosynthesis^9–11^. This competition between substrates ultimately would result in the reduction of RA levels to teratogenic levels (Fig. 8). Biosynthesis of RA requires two oxidation steps, the first from ROL to RAL is rate limiting based on enzymatic considerations^9,38^, while the second oxidation of RAL to RA is limited by enzyme availability^39^. In recent years, it has become evident that during early embryogenesis, the oxidation of ROL is performed mainly by members of the SDR family like, RDH10 and RDH2^23,24,40,41^. On the other hand, EtOH is oxidized by middle-chain ADH enzymes^7,42,43^. Taking advantage of enzyme family specific inhibitors, we obtained additional evidence of channeling of the EtOH and ROL to different enzyme families for oxidation. Inhibition of the SDR activity did not prevent EtOH from affecting the expression of RA-regulated genes. In contrast, inhibition of enzymes of the ADH family efficiently prevented the RA-antagonizing activity of EtOH. These results show that EtOH requires ADH-mediated oxidation to achieve its RA-reducing activity and, it does not compete for the SDR enzymes during early embryogenesis (Fig. 8).

AcAL, a highly toxic organic compound, induces a wide range of pharmacological, behavioral, and carcinogenic responses in humans^44,45^. AcAL can form adducts with DNA, proteins, and lipids and promote the formation of reactive oxygen species^7,46,47^. The requirement for an ADH activity to trigger the RA-inhibiting effect of EtOH places the focus on AcAL as the mediator of this effect. Further support was obtained by treating embryos with AcAL or EtOH at physiological concentrations. The set of developmental malformations obtained from both treatments were indistinguishable, suggesting that both compounds affect the same set of developmental processes, and EtOH is being oxidized to AcAL for the teratogenic effect to be induced. Together, all these observations shift the focus from EtOH to AcAL as the main teratogenic compound from alcohol exposure competing for the second oxidation step in RA biosynthesis (Fig. 8).

For the competition model to take place, AcAL should function under physiological conditions, as an efficient substrate for enzymes with RALDH activity. In previous studies, we provided evidence showing that in *Xenopus* embryos the onset of gastrulation is the most alcohol sensitive developmental stage when the most severe FAS-like malformations are induced^18^. Interestingly, RA signaling in vertebrate embryos has been shown, to begin with the onset of gastrulation, in the region of the embryonic organizer^18,48,49^. At these stages, most components of the RA biosynthetic and signaling network are apparently present and await the RALDH-mediated oxidation of RAL to RA to complete and activate the pathway^48,50–52^. The biosynthesis of RA and the onset of signaling correlate with the start of expression of the *Raldh2* gene in vertebrate embryos^20,53–56^. In agreement, mutation of the *Raldh2* gene results in the earliest embryonic lethality among mutations in members of the RA metabolic network^57^. This observation suggests that the enzyme encoded by this gene is crucial for gastrulation and early neurulation^20,53,55,56^. All these observations place the focus on the RALDH2 enzyme as one of the earliest targets of the alcohol exposure. For this reason, we characterized in more detail the RALDH2 enzyme and in particular the human ortholog to try and understand its role in the induction of FASD^35^.

Focusing on hRALDH2, we could show that the effects of EtOH on RA-regulated genes can be rescued by increasing the level of RALDH2 activity in the embryo. In the original model, EtOH would inhibit RAL formation^9^. Then, according to the model, the addition of RALDH2 would have no effect as no substrate would be available. The restricted availability of RALDH2 or RAL-oxidizing activity was experimentally demonstrated^50^, supporting the labile condition of RA biosynthesis at the onset of gastrulation. At these stages, *Raldh2* transcript accumulation is just beginning, and enzyme availability is low. In agreement, our results show that the increase in RALDH2 activity compensates for the competition by AcAL and allows sufficient oxidation of RAL to RA, supplementing the limiting availability of this enzyme.

The central aspect of the RALDH2 study was to demonstrate that AcAL is an efficient substrate for this enzyme and as such competes with RAL. Characterization of hRALDH2 for several RAL isomers known to be present *in vivo*, *all-trans, 9-cis*, and *13-cis*, revealed similar enzymatic efficiencies^35^. Taking advantage of the same *in vitro* conditions, we determined the enzymatic parameters of hRALDH2 when acetaldehyde was provided as the substrate. Our results show that the K_m_ measured for acetaldehyde (8.47 µM) is about 2-fold lower than the K_m_ when *all-trans* RAL is the substrate while the V_max_ for both substrates is similar. These results show that hRALDH2 can efficiently use AcAL as a substrate and it has a preference for it over RAL under our experimental conditions. The demonstration that acetaldehyde is a substrate of hRALDH2 strongly supports the competition model between AcAL and RAL for the available RALDH as an important component of the etiology of FASD. Then detoxification of the EtOH ingested can induce a teratogenic reduction in RA levels through the competition for the available RALDH activity. For a direct demonstration of the competition between acetaldehyde and retinaldehyde and the resulting inhibition of RA production, the *in vitro* reaction products were analyzed by HPLC. This type of analysis demonstrated a concentration-dependent inhibitory effect of AcAL on the production of RA from RAL when both substrates are present together. This result further supports the competition for hRALDH2 and the resulting reduction in RA production.

Acetaldehyde has been studied as a substrate for several vertebrate RALDH enzymes. This substrate has been studied with RALDH1 from human, rat and *Xenopus* and the RALDH2 from rat. For those enzymes, the K_m_ values reported range from 15 µM to 2500 µM^36,58–61^. Kinetic analysis of the human RALDH1 revealed a lower K_m_ when acetaldehyde was used in the 0 to 50 µM range, and it increased dramatically when higher AcAL concentrations were used. These results led to the proposal that the RALDH enzymes bind their substrates cooperatively^62^. For hRALDH2, we measured the K_m_ with AcAL concentrations in the 0 to 50 µM range, which includes the physiological range in human blood (up to 20 µM)^27–31^. The range of K_m_ values measured for AcAL for the different RALDH enzymes could reflect real enzymatic differences, or it could be a result of the cooperative behavior and the reaction conditions.

Other acetaldehyde-oxidizing enzymes could be performing the removal of this toxic compound. Analysis of the expression of *Alhd2* during early embryogenesis identified maternal transcripts whose levels decrease markedly towards the onset of gastrulation. All other genes studied, *Raldh1* (*Aldh1A1*), *Raldh2* (*Aldh1A2*), *Raldh3* (*Aldh1A3*), were expressed at late blastula by zygotic transcription, while *Aldh1B1* transcripts were undetectable at these stages. By early/mid-gastrula, *Raldh2* is the most abundant gene expressed among those studied, while the *Aldh2* transcripts represent less than 10% of the *Aldh* transcripts detected. These observations suggest that RALDH2 is placed to perform most of the RA biosynthesis in the embryo. In the case of EtOH exposure, RALDH2 is the most abundant enzyme capable of efficient AcAL oxidation at the expense of RA.

The inhibition of the hRALDH2 activity by AcAL is the biochemical mechanism of the competition model by diverting the early embryonic RALDH activity from RAL oxidation and RA production. The competition of acetaldehyde for the available RALDH2 activity was previously suggested from EtOH rescue experiments using ROL and RAL supplementations^18^. Also, increasing hRALDH2 enzyme levels, lowered the competition between acetaldehyde and the endogenous substrate, retinaldehyde, and restored RA signaling by supporting its production in the presence AcAL. Alternatively, increasing the available RAL also rescues the effects of AcAL. Then, biochemically, the competition mechanism is explained by the demonstration that AcAL, is a substrate of RALDH2 *in vivo* and *in vitro*, and the K_m_ for this substrate is lower than that of RAL, making a preferred substrate.

In the context of FASD, acetaldehyde levels can reach 2–20 µM in the mother’s blood as a result of alcohol intake^27–31^. During pregnancy, the mother performs the main alcohol detoxification. Among the enzymes involved, ALDH2 is considered a central player in the liver, although additional enzymes are involved^63^. The remaining AcAL then crosses the placenta^64,65^, and once it reaches the embryo, it competes and hampers RAL oxidation resulting in reduced RA signaling with teratogenic outcomes. Multiple maternal alleles encoding alcohol and aldehyde dehydrogenases have been identified as risk-increasing or protective for FASD^42^. These observations support a genetic component for FASD, as genetic polymorphisms might affect enzymatic activity levels and impact on the efficiency of ethanol detoxification.

According to the proposed mechanism, the competition for the RALDH activity by AcAL results in a reduction in RA levels. This teratogenic mechanism is also the basis of the Vitamin A Deficiency Syndrome, and in agreement, the developmental malformations in both diseases are similar^66^. A similar RA signaling reduction has also been proposed for the Matthew-Wood, Smith-Magenis, and DiGeorge/VeloCardioFacial syndromes and they exhibit reminiscent developmental malformations^67–69^. Alcohol is also recognized as a risk-factor for several different types of tumors^70^. It has been shown that acetaldehyde is the active carcinogenic metabolite of EtOH among alcoholics^45^. The tumor-promoting mechanism of EtOH could involve the same biochemical effect on RA levels as this signaling pathway performs numerous regulatory roles beyond embryonic development. RA signaling is involved in tissue homeostasis with anti-proliferative, tumor suppressor roles extending to multiple tissues like prostate, breast, and colon. In some types of cancer, the analysis of RA mediators or target genes are used as biomarkers of tumor prognosis^70^. Also in these cases, the contribution of EtOH to the disease progression could involve a reduction in RA signaling with its tumor-promoting effect. Therefore, our study suggests new aspects of alcohol as a detrimental compound where RA is crucial for developmental processes or tissue homeostasis.

## Methods

### Embryo staging and treatments

For *Xenopus laevis* microinjection of plasmids, 2- to 4-cell embryos were injected radially (4 times) in 1X modified Barth’s saline with HEPES (MBSH) and incubated in 0.1X MBSH. For treatment, embryos were incubated until st.8.5 and then treated as indicated. Embryos were staged based on morphological landmarks according to Nieuwkoop and Faber^71^.

Retinoids and HPLC grade solvents were purchased from Sigma-Aldrich (St. Louis, Missouri): *all-trans* Retinal (RAL), *all-trans* Retinoic acid (RA), Dimethyl sulfoxide (DMSO), Acetaldehyde, 4-Diethylaminobenzaldehyde (DEAB), Chloral Hydrate (CH), carbenoxolone (CBX), 4-methylpyrazole (4MP), acetonitrile, hexane and methanol. Stock solutions of RAL, RA, and DEAB, were prepared in DMSO. Experiments were performed in accordance with the relevant guidelines and regulations of the Institutional Ethics Committee of The Hebrew University after their approval and under their supervision.

### RALDH2 enzyme assays

Expression and purification of the human RALDH2 protein were according to Shabtai *et al*.^35^. hRALDH2 enzymatic activity was monitored either by spectrophotometer or by high-pressure liquid chromatography (HPLC). The RALDH2 enzymatic reaction was performed as previously described^35^. Standard reactions were performed at 37 °C in 50 mM Hepes/K^+^ pH 8.5 buffer containing 2 mM MgCl2, 2 mM DTT, 150 mM KCl and 1 mM EDTA (final volume 200 µl). Protein content in the reaction mixtures was 1 µg. Reactions were initiated by addition of the substrate: retinoids or acetaldehyde (1 to 100 µM) in DMSO and a constant NAD^+^ 1 mM concentration.

For spectrophotometric determination of the hRALDH2 activity, the reaction was monitored at 340 nm for the overall increase in absorbance of produced NADH. A global extinction coefficient of 2,820 M^−1^s^−1^ was experimentally determined by measuring the increase in absorbance of increasing NADH under our reaction conditions. Initial rate measurements were carried out at 37 °C on a TECAN Infinite F200Pro spectrophotometer sampling the OD every 10 sec for a total of 30 min. The kinetic constants of RALDH2 were obtained by plotting saturation curves under conditions in which enzyme activity was linear with respect to protein concentration and reaction time, and fitting data from the saturation curve to Michaelis–Menten with the Prism software (Graph Pad Software Inc., San Diego, CA).

For HPLC analysis of RA production, the enzymatic reaction was stopped by adding 400 µl of acetonitrile. Methanol was added after sample evaporation and subjected to HPLC analysis. HPLC was performed based on Frolik *et al*.^72^. Shortly, run protocol: 50 µl/sample were injected and run at 700 psi, 1.1 ml/min, acetonitrile:1% ammonium acetate 80:20 was used as the developing solvent. Instruments: Waters 717 autosampler, Waters 996 Photodiode Array Detector, Waters 600 pump, Waters 600 controller. Column: Spherisorb ODS2 Column, 80 Å, 5 µm, 4.6 mm × 250 mm. Total RA production was measured after 25 min, n = 4 in duplicate.

### Whole mount ***in situ*** hybridization

The whole mount *in situ* hybridization was performed as described previously^73^. Probe information: HoxB3^74^, En2^75^, Gsc^18^. Comparisons of distances of anatomical markers and structures were analyzed with the ImageJ software package.

### Quantitative real-time RT-PCR (qPCR)

Total RNA from embryos was extracted with Aurum™ Total RNA Mini Kit with DNase (Bio-Rad). Each experiment was repeated at least 3 times and involved at least 5-embryos/samples. cDNA was synthesized using iScript cDNA Synthesis kit (Bio-Rad). qPCR was done on an CFX384 Touch™ Real-Time PCR Detection System (Bio-Rad) using the iTaq™ Universal SYBR Green Kit (Bio-Rad). Results were calculated with the ∆∆*CT* method^76^, and the primers used were:

*Gapdh*: 5′GCTCCTCTCGCAAAGGTCAT, 5′GGGCCATCCACTGTCTTCTG;

*HoxB1*: 5′TCCCCCTCCAACAACAAACC, 5′TTGCCCCAGTGCCAATGAC;

*HoxA1* 5′CATCGCCTCGTCTGTGGT, 5′GTCAGGTCCGTATGAATGGTG;

*HoxB4*: 5′CCAAGGATCTGTGCGTCAA, 5′GCAGGATGGAGGCGAACT;

*Cyp26A1*: 5′CGATTCCTCAAGGTTTGGCTTCA, 5′ATTTAGCGGGTAGGTTGTCCACA;

*Rdh10*: 5′TTTGAAGCTGTGGTCTGCAT, 5′GCCTGTTTCCTTTGAGCAAT;

*Dhrs3*: 5′CAGGCGCAAGAAATCCTAAG, 5′CAAAGGCCACGTTACAGGAT;

*Raldh1*: 5′GAACTTTCCGTTGTTGATGT, 5′GATAGCAGTCAGTGGAGTTTG;

*Raldh2*: 5′ATGTTTGCCTGGAAGATTGC, 5′GAGAGCAGTGAGCGGAGTCT;

*Raldh3*: 5′TAAAGCCCTGTCTGTTTCT, 5′CATACTCTCCAAGTTCCCTT;

*Aldh2*: 5′AGGCTGGGCTGACAAATG, 5′ CACACACTCCGACAGGTTCA;

*Aldh1B1*: 5′GCAATCCCTTTGACCTGGA, 5′GCACCTTCTGTTTTACCGCTTT;

*Chordin: 5′ACTGCCAGGACTGGATGGT, 5′ GGCAGGATTTAGAGTTGCTTC*

### Statistics

All statistical comparisons were carried out using Prism software (Graph Pad Software Inc., San Diego, CA). Results are given as the mean ± standard error of the mean (SEM). Tests used were the Two-sample *t*-test for proportions or nonlinear regression plotting Michaelis–Menten constants, as appropriate. Differences between means were considered significant at a significance level of p < 0.05.

### Data availability

All data generated or analyzed during this study are included in this published article (and its Supplementary Information files).

## Electronic supplementary material

Supplementary Figures
