# Supplementary material for: Acetaldehyde inhibits retinoic acid biosynthesis to mediate alcohol teratogenicity
Source: Sci Rep. 2018 Jan 10;8:347. doi: 10.1038/s41598-017-18719-7 (PMC5762763; doi:10.1038/s41598-017-18719-7)
Supplement: Supplementary file 1 — Supplementary Figures [file 41598_2017_18719_MOESM1_ESM.pdf]

Acetaldehyde inhibits retinoic acid biosynthesis to mediate alcohol teratogenicity

Yehuda Shabtai[1], Liat Bendelac[1], Halim Jubran[2], Joseph Hirschberg[2] and Abraham Fainsod[1]

[1]Department of Developmental Biology and Cancer Research, Institute for Medical Research Israel-Canada, and [2]Department of Genetics, The Alexander Silberman Institute of Life Sciences, Faculty of Science, The Hebrew University of Jerusalem, Jerusalem, Israel.

Supplementary Figures

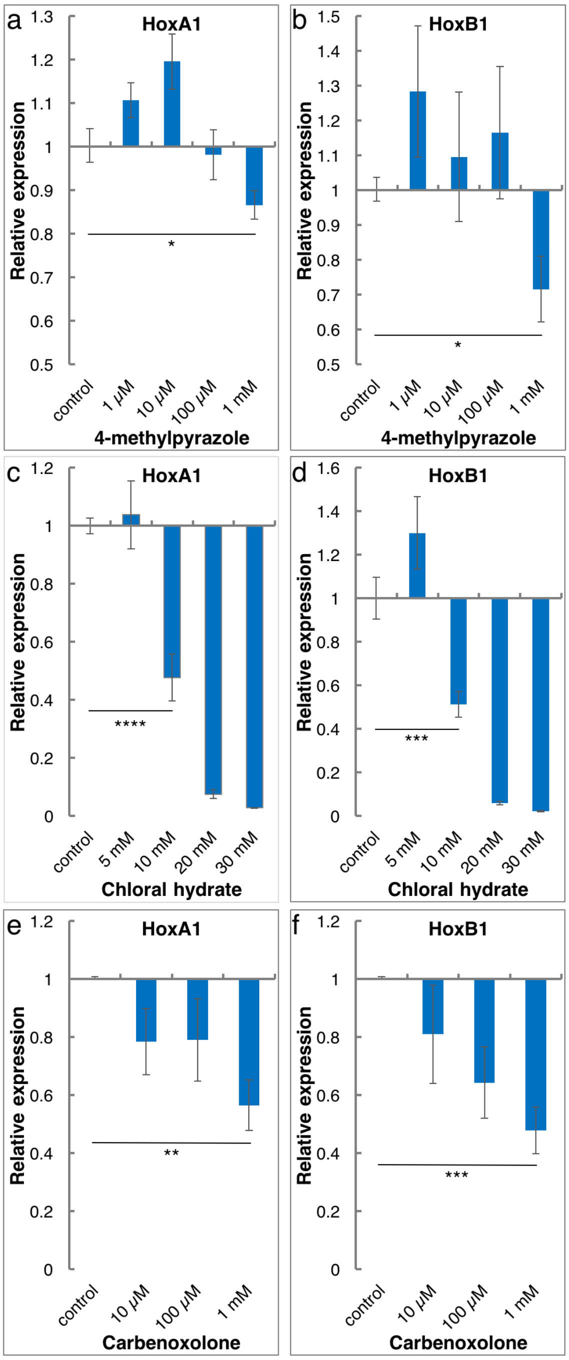

Supplementary Figure S1. Inhibition of the ADH and SDR enzymes in the early embryo. Embryos were treated with increasing amounts of (**a**,**b**) 4-methylpyrazole (1-1000 μM), (**c**,**d**) chloral hydrate (5-30 mM), or (**e**,**f**) carbenoxolone (10-1000 μM). The effect of these treatments on the expression on the RA-regulated genes, *HoxA1* and *HoxB1*, was monitored by qPCR. The p-value is shown for the concentration chosen for subsequent experiments. n=3, The values denote mean ± SEM. P values - *p<0.05; **p<0.01; ***p<0.001; ****p<0.0001.

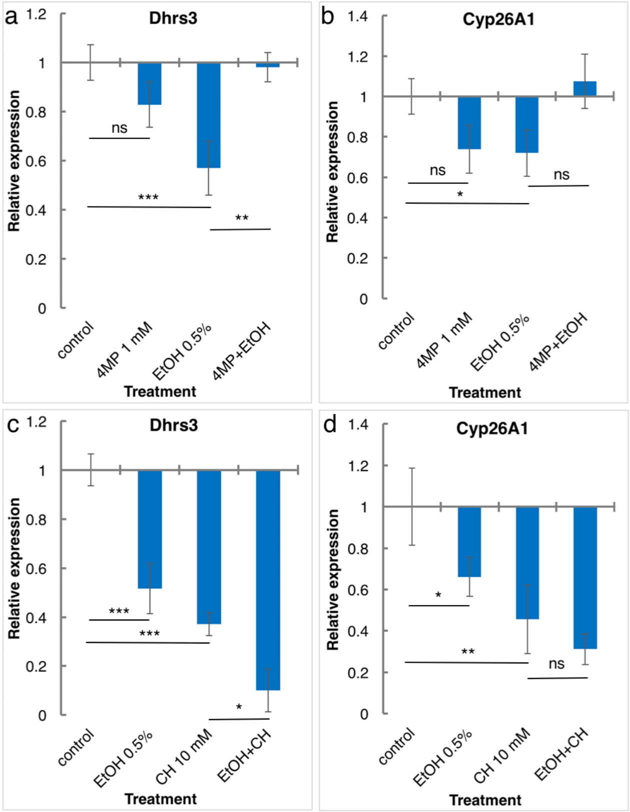

Supplementary Figure S2. Ethanol-dependent retinoic acid inhibition requires middle-chain alcohol dehydrogenases. The enzymatic requirements for EtOH to inhibit RA biosynthesis were studied using inhibitors of the middle-chain alcohol dehydrogenases (ADH), 4-methylpyrazole (4MP), or the short-chain dehydrogenase/reductases, chloral hydrate (CH). Late blastula stage embryos were treated with EtOH alone or in combination with 4MP (**a**,**b**) or CH (**c**,**d**). The effect on RA signaling was determined by monitoring the expression level of the known RA-regulated genes, *Dhrs3* (**a**,**c**) and *Cyp26A1* (**b**,**d**) during early gastrula stages. n=3, The values denote mean ± SEM. P values - *p<0.05; **p<0.01; ***p<0.001; ns, not significant.

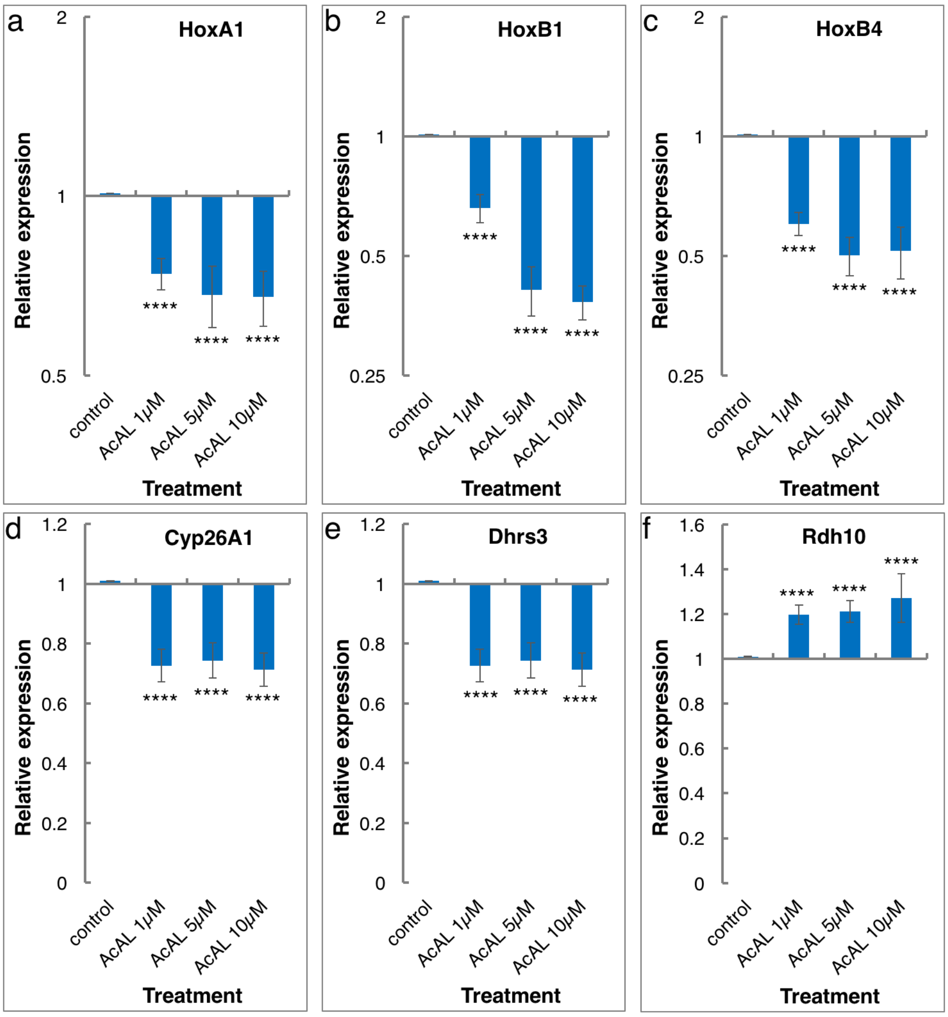

Supplementary Figure S3. Acetaldehyde affects the expression of retinoic acid-regulated genes. Embryos were treated with AcAL (1-10 μM) from late blastula (st. 8.5), and the effect on the expression of RA-regulated genes was determined by qPCR at st. 10.5. The genes studied were; *HoxA1* (**a**), *HoxB1* (**b**), *HoxB4* (**c**), *Cyp26A1* (**d**), *Dhrs3* (**e**), and *Rdh10* (**f**). n=3, The values denote mean ± SEM. P values - ****p<0.0001.

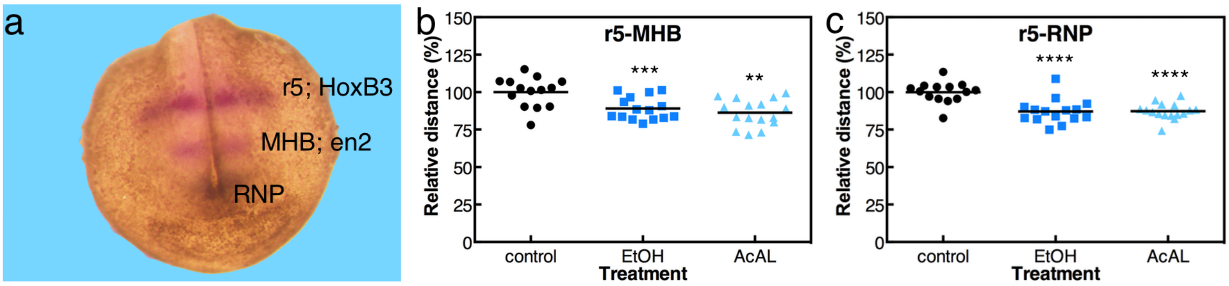

Supplementary Figure S4. Acetaldehyde and ethanol modify the brain region. Embryos treated with AcAL (1 μM) or EtOH (0.5%) from late blastula were allowed to develop to st. 18. (**a**) The embryos were hybridized *in situ* with *HoxB3* and *en2* specific probes to mark rhombomere 5 (r5) and the midbrain/hindbrain boundary (MHB) respectively. The distance between r5 and the MHB and between r5 and the rostral end of the neural plate (RNP) was measured. Comparison in the r5-MHB (**b**) and r5-NRP (**c**) distances between AcAL and EtOH-treated embryos. The ratio of the relative change of the r5-MHB and r5-NRP distances for each treatment is shown. n=45, P values - **p<0.01; ***p<0.001; ****p<0.0001.

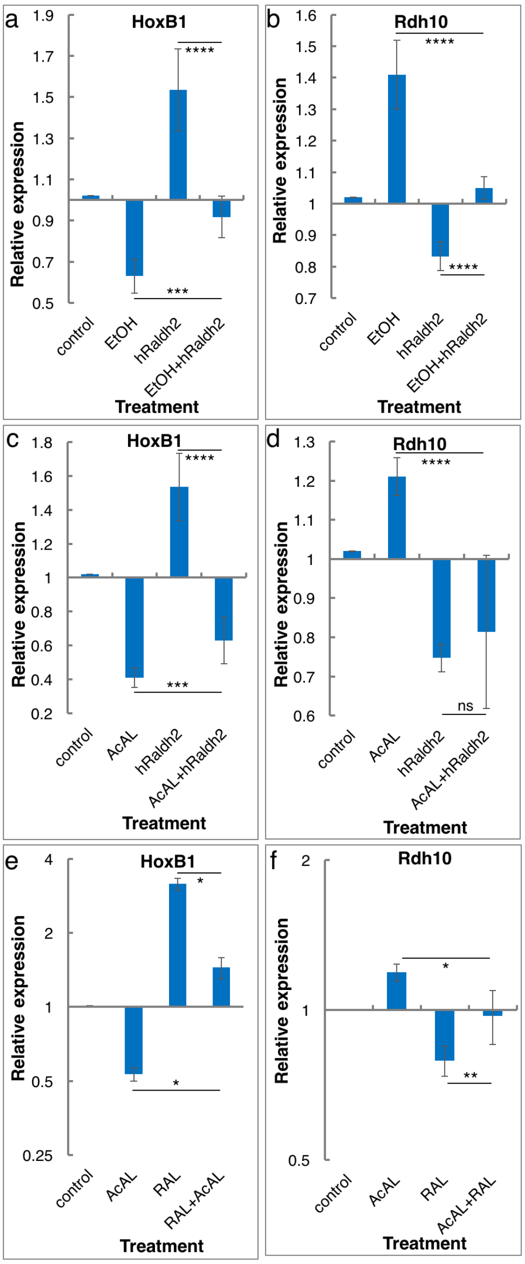

Supplementary Figure S5. Acetaldehyde competes for the human RALDH2. The effect of ethanol and acetaldehyde on RALDH2 activity was studied in manipulated embryos. Embryos treated with AcAL (5 μM), EtOH (0.5%) or RAL (1 μM) were injected with plasmid encoding hRALDH2. Treatments were initiated during late blastula, and RNA samples were prepared during early/mid-gastrula. The effect of the combined treatments was determined by analyzing the response of the RA-regulated genes, *HoxB1* (**a**,**c**,**e**), and *Rdh10* (**b**, **d**,**f**) by qPCR. (**a**,**b**) Overexpression of hRALDH2 together with EtOH treatment. (**c**,**d**) AcAL treatment together with hRALDH2 overexpression. (**e**,**f**) Combined treatment with AcAL and RAL. n=3, The values denote mean ± SEM. P values - *p<0.05; **p<0.01; ***p<0.001; ****p<0.0001; ns, not significant.
